# LRIG2 is a growth suppressor of Hec-1A and Ishikawa endometrial adenocarcinoma cells by regulating PI3K/AKT- and EGFR-mediated apoptosis and cell-cycle

**DOI:** 10.1038/s41389-017-0019-1

**Published:** 2018-01-23

**Authors:** Dae-Shik Suh, Si Eun Park, Hanyong Jin, Kangseok Lee, Jeehyeon Bae

**Affiliations:** 1 Division of Gynecologic Oncology, Department of Obstetrics and Gynecology, Asan Medical Center, University of Ulsan College of Medicine, Seoul, 05505 Korea; 2 School of Pharmacy, Chung-Ang University, Seoul, 06974 Korea; 3 Department of Life Science, Chung-Ang University, Seoul, 06974 Korea

## Abstract

Although endometrial cancer is the most common type of gynecological malignancy in developed countries, its molecular etiology is not well understood. Leucine-rich repeat and immunoglobulin-like domain 2 (LRIG2) is an evolutionarily conserved gene, but its functions in the endometrium are unknown. In this study, we found that LRIG2 is highly downregulated in endometrial adenocarcinoma patients and that it functions as a tumor suppressor. LRIG2 induced the mitochondrion-mediated apoptotic pathways by regulating stoichiometric balance among BCL-2 family proteins, whereby pro-survival members, MCL-1 and BCL-xL, were downregulated and pro-apoptotic BAK and BAX were upregulated. LRIG2 also inhibited proliferation of the Hec-1A and Ishikawa endometrial adenocarcinoma cells by upregulating p21. LRIG2 induced BAX- and BAK-dependent cell death that was efficiently prevented by MCL-1 overexpression. Furthermore, we found that LRIG2 unexpectedly phosphor-activates phosphoinositide 3-kinase (PI3K)/AKT and epidermal growth factor receptor (EGFR), which are conventionally accepted as survival signaling cues in diverse types of cancer. We observed that PI3K/AKT and EGFR serve as key kinases that have roles as growth suppressors of Hec-1A endometrial cancer cells by mediating the LRIG2-induced modulation of the BCL-2 family of proteins and p21. In vivo delivery of antisense DNAs against LRIG2 promoted the Hec-1A endometrial tumor growth in a xenograft mouse model, and immunoblotting of these tumor extracts showed consistent modulation of AKT, EGFR, the BCL-2 family members, and p21. Thus, our results demonstrated that LRIG2 is a growth suppressor of endometrial adenocarcinoma cells.

## Introduction

Endometrial cancer is the most frequently occurring gynecological cancer in developed countries, and its rapidly increasing incidence causes great concern^1^. Endometrial carcinoma is the major uterine cancer comprising 80–90% of cases^2^. Although the molecular mechanisms underlying development of endometrial carcinoma are not well understood, prolonged exposure to and high levels of estradiol is a known risk factor for this cancer^3,4^.

The protein family containing leucine-rich repeats and immunoglobulin-like domains (LRIG) is an evolutionarily conserved group of proteins with a single transmembrane domain^5,6^. In humans, three homologs, LRIG1, LRIG2, and LRIG3, have been described. LRIG2 shares 41 and 54% of amino acid identity with LRIG1 and LRIG3, respectively^7–9^. At present, functions of the human LRIG family remain enigmatic^6^, as only scarce relevant information is available. Comparative analysis of *LRIG2* mRNA abundance in human organs showed its predominant expression in the female reproductive system, especially in the uterus and ovaries^8^. This observation raises the possibility that LRIG2 could have important roles in these organs, but its functions in these organs have not been reported.

Cellular homeostasis in the body is maintained by controlling cell death and proliferation; dysregulation of these processes leads to a wide spectrum of disorders, including cancers^10^. The BCL-2 family of proteins are evolutionarily conserved central regulators of apoptosis; they comprise counteracting members, which are either pro-survival or pro-apoptotic proteins^11^. The BCL-2 subfamily of pro-survival proteins include MCL-1, BCL-2, BCL-xL, BCL2A1, and BCL-w. The pro-apoptotic subfamily members include BAX, BAK, BAD, BIM, and BID^11^. The BCL-2 family of proteins are located at the outer mitochondrial membrane and control cellular apoptosis by homodimerization or heterodimerization among members. Delicate, competing equilibriums between these proteins determine a cell’s fate^12^. Upon death signaling, BAX and BAK, two death-effector molecules, oligomerize, leading to mitochondrial outer membrane permeabilization (MOMP) and release of apoptotic molecules, including cytochrome *c*, which subsequently activates caspases^13^. On the other hand, regulation of cell-cycle progression by cyclin-dependent kinases (CDKs) controls cellular proliferation^14^. p21 (CIP1, WAF1), which is encoded by the cyclin-dependent kinase inhibitor 1A (CDKN1A) gene, is a potent cell-cycle regulator that inhibits the functions of cyclin–CDK1, cyclin–CDK2, cyclin–CDK4, and cyclin–CDK6 complexes, thus arresting cell-cycle progression^15^.

In this study, we demonstrate, for the first time, that LRIG2, a previously unreported growth suppressor, inhibits the growth of endometrial adenocarcinoma by inducing apoptosis and inhibiting cell proliferation. Moreover, we report that LRIG2 is downregulated in endometrial adenocarcinoma tissues of patients. We also describe the signaling pathways underlying the tumor-suppressive effects of LRIG2.

## Results

### LRIG2 induces mitochondrial apoptotic cell death

**Fig. 1:**
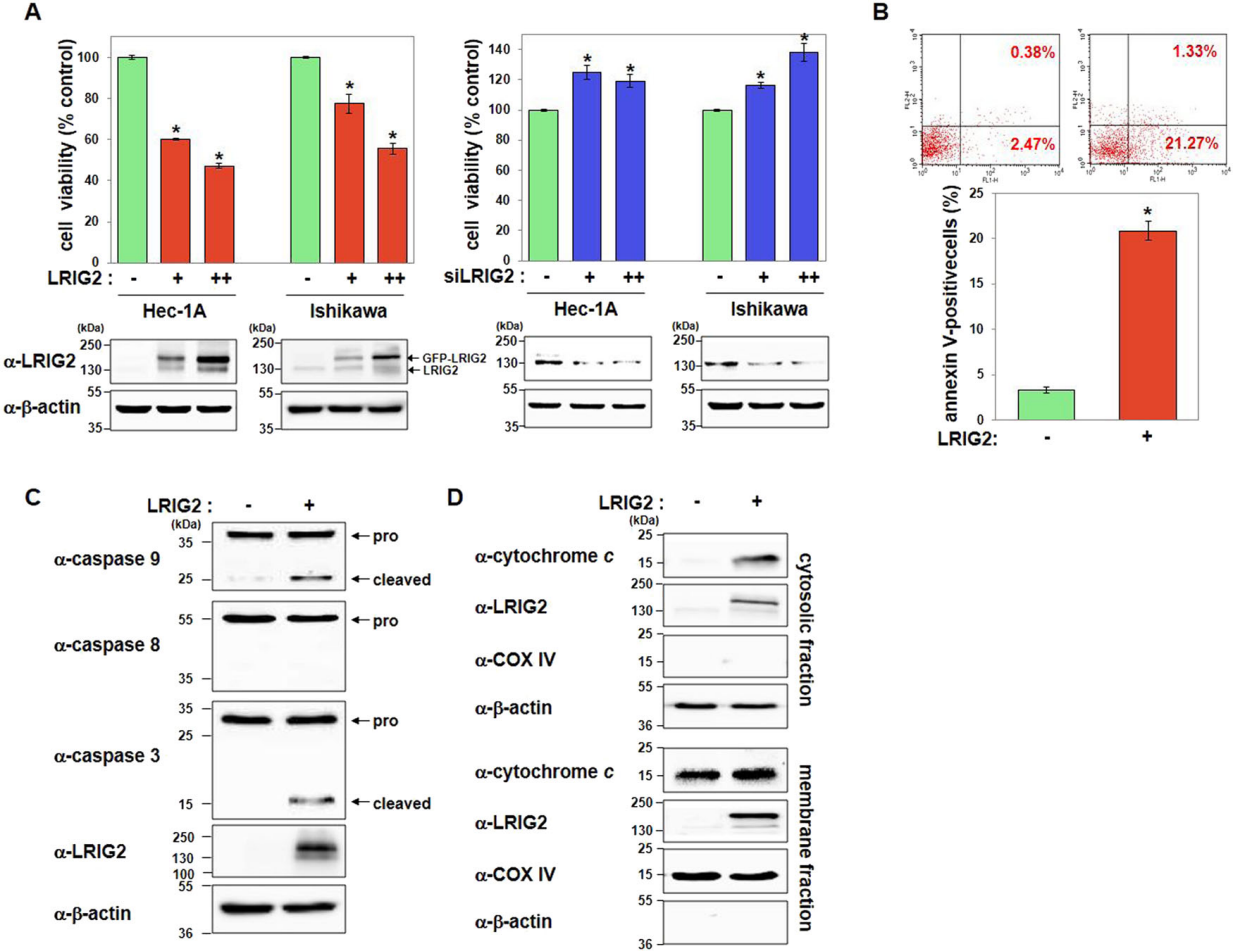
LRIG2 induces mitochondrion-mediated apoptosis of endometrial carcinoma cells. **a** Hec-1A and Ishikawa cells were transfected with plasmids encoding LRIG2–GFP (50 or 100 ng) or siRNA #1 (100 or 200 nM) against LRIG2. As controls, either an empty vector or scrambled siRNAs were transfected. Cell viability was measured 24 h after transfection. **b** LRIG2–GFP-overexpressing Hec-1A cells were analyzed to detect the annexin-V-positive apoptotic cells by flow cytometry. Representative scatter plots (top) and quantified results (bottom) are shown. **c** Cell lysates of LRIG2–GFP-overexpressing Hec-1A cells were subjected to immunoblotting for caspases using the indicated antibodies. **d** Subcellular fractionation was performed using Hec-1A cells after LRIG2–GFP transfection. Cytosolic release of cytochrome *c* was determined by western blotting. Efficient fractionation was confirmed by immunoblotting of β-actin and COX IV. All quantified results are mean ± SEM of three independent experiments performed in triplicates (*p < 0.05)

To determine cellular functions of LRIG2, we performed cell-viability assays using endometrial adenocarcinoma cell lines. Overexpression of either GFP-tagged LRIG2 or LRIG2-induced cell death, whereas LRIG2 knockdown using two independent siRNAs promoted cell survival in both Hec-1A and Ishikawa cells (Fig. 1a; Supplementary Figures S1a and b). The number of annexin-V-positive apoptotic Hec-1A cells was significantly increased following ectopic expression of LRIG2 (Fig. 1b). LRIG2 activated the initiator caspase 9 and the effector caspase 3, whereas caspase 8 cleavage was not readily detected (Fig. 1c). LRIG2 was expressed in both the membrane and cytosolic fractions of endometrial cancer cells and caused release of mitochondrial cytochrome *c* into the cytosol, indicating that LRIG2-induced mitochondrial apoptosis (Fig. 1d).

### BCL-2 family of proteins mediate LRIG2-induced cell death

**Fig. 2:**
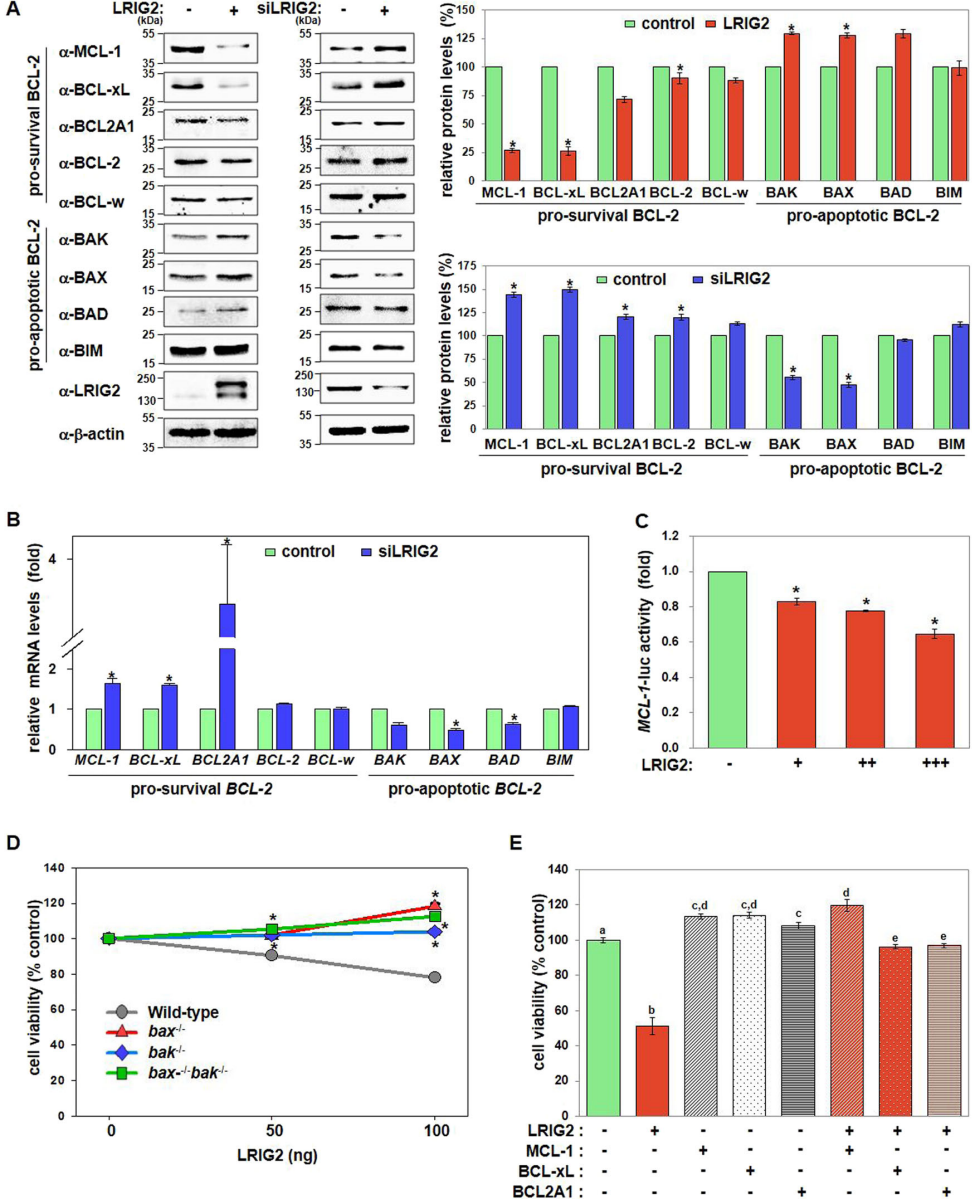
Cell-death activity of LRIG2 is mediated by regulating the stoichiometric balance among BCL-2 family of proteins. **a** LRIG2 was either overexpressed or silenced as described in the legend of Fig. 1a. Representative immunoblotting results and quantified data of three independent experiments are presented. **b** Changes in the mRNA levels of the *BCL-2* family in LRIG2-depleted Hec-1A cells were quantified by real-time RT-PCR. **c** Human *MCL-1* promoter activation by LRIG2 was measured by luciferase assays 24 h after transfection using Hec-1A cells transfected with increasing amounts of plasmids (50, 100, or 200 ng) encoding LRIG2. **d** Wild-type (WT) and *bak*^*−/−*^, *bax*^*−/−*^, and *bax*^*−/−*^/*bak*^*−/−*^ MEF cells were transfected with the indicated amounts of LRIG2 plasmid, and cell viability was measured 24 h after transfection. **e** Hec-1A cells were transfected with MCL-1, BCL-xL, or BCL2A1 with or without LRIG2. Cell viability was measured after 24 h. All quantified results are mean ± SEM of three independent experiments performed in triplicates. Asterisks or different letters indicate statistically significant values (*p* < 0.05)

Next, we investigated the underlying mechanisms by which LRIG2 exerted pro-apoptotic effects. Because the LRIG2-induced cell death involves the mitochondrial intrinsic apoptosis pathway (Fig. 1c), we determined whether LRIG2 regulates the BCL-2 family. Ectopic expression of LRIG2 downregulated the pro-survival BCL-2 family members, including MCL-1, BCL-xL, BCL2A1, and BCL-2, whereas the pro-apoptotic BCL-2 family members, such as BAK, BAX, and BAD, were upregulated by LRIG2 in endometrial cancer cells (Fig. 2a). Conversely, LRIG2 knockdown exhibited opposite effects on the expression of these BCL-2 members (Fig. 2a). In general, LRIG2-mediated effects on the expression of BCL-2 protein family involved their transcriptional regulation, as the mRNA levels of *BCL-2* members were also modulated following LRIG2 silencing (Fig. 2b). LRIG2-mediated transcriptional regulation of *BCL-2* genes was further confirmed by demonstrating that LRIG2 repressed the human *MCL-1* promoter activity (Fig. 2c).

In addition, roles of BAK and BAX in LRIG2-induced cell death were determined using *bax*^−/−^, *bak*^−/−^, and *bax*^−/−^*bak*^−/−^ MEF cells. LRIG2 also caused death of the wild-type (WT) MEF cells (Fig. 2d). In contrast, LRIG2 failed to kill *bax*^−/−^, *bak*^−/−^, and *bax*^−/−^*bak*^−/−^ MEF cells (Fig. 2d). In addition, expression of MCL-1 completely blocked the LRIG2-induced cell death, whereas overexpression of BCL-xL or BCL2A1 partially prevented the LRIG2 response (Fig. 2e), implying that MCL-1 is a crucial mediator of LRIG2-induced apoptotic activity.

### LRIG2 induces p21-mediated cell-cycle arrest

**Fig. 3:**
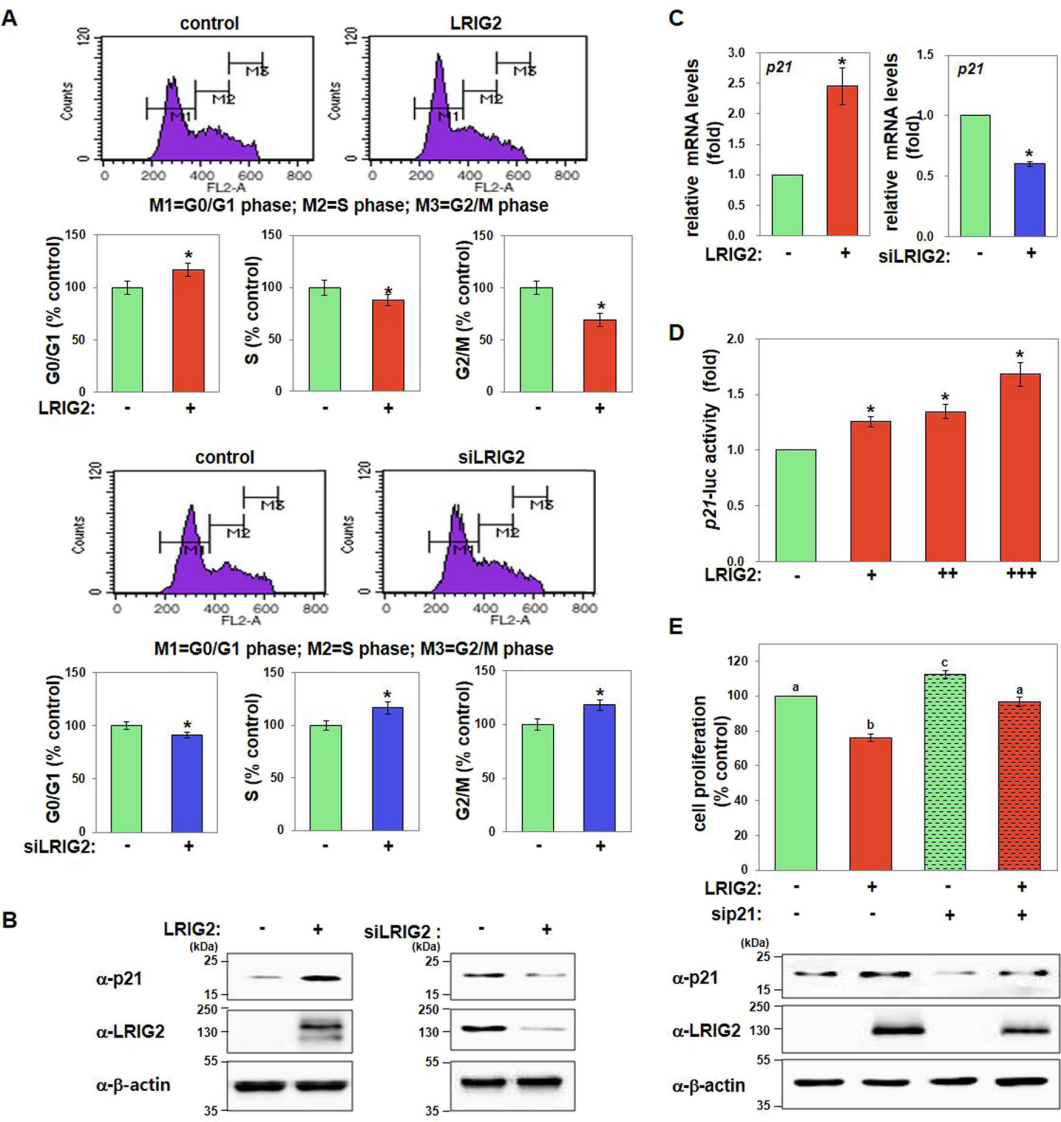
LRIG2 inhibits cell-cycle progression in endometrial carcinoma, and this is mediated by p21. **a** LRIG2-induced effects on cell-cycle progression were assessed in LRIG2–GFP-overexpressing or LRIG2-depleted Hec-1A cells. Representative histograms and quantified cell populations in the G0/G1, S, and G2/M phases are shown. **b**, **c** The same cells shown in **a** were used for immunoblotting **b** or real-time RT-PCR **c** to detect p21. **d** Human *p21* promoter activation by LRIG2 was determined by luciferase assays 24 h after transfection in Hec-1A cells transfected with increasing amounts of plasmid (50, 100, or 200 ng) encoding LRIG2–GFP. **e** Hec-1A cells were transfected with control siRNAs or p21-specific siRNAs for 24 h. Cells were then further transfected with either an empty vector or the LRIG2 plasmid, and cell proliferation was assayed after 24 h. Effective p21 knockdown was confirmed by immunoblotting. All quantified results are mean ± SEM of three independent experiments performed in triplicates (*p < 0.05). Different letters indicate statistically significant values (*p* < 0.05)

We also assessed the effect of LRIG2 on cell-cycle progression in endometrial carcinoma cells using flow cytometry. Figure 3a shows that LRIG2 overexpression increased the cell population in the G_0_/G_1_ phase with a concomitant decrease in the number of cells in the S and G_2_/M phases; opposite effects were observed following LRIG2 knockdown. Western blotting of LRIG2-overexpressing of LRIG2-silenced cells showed that p21 was markedly upregulated or downregulated, respectively (Fig. 3b). Real-time RT-PCR results indicated that *p21* mRNA levels also increased or decreased by overexpression or knockdown of LRIG2, respectively (Fig. 3c). In addition, luciferase reporter analysis of the human *p21* promoter confirmed the transcriptional activation of *p21* by LRIG2 (Fig. 3d). Moreover, LRIG2-induced inhibition of cell proliferation was attenuated upon p21 knockdown (Fig. 3e).

### The PI3K-mediated AKT and EGFR pathways are involved in growth inhibition by LRIG2

**Fig. 4:**
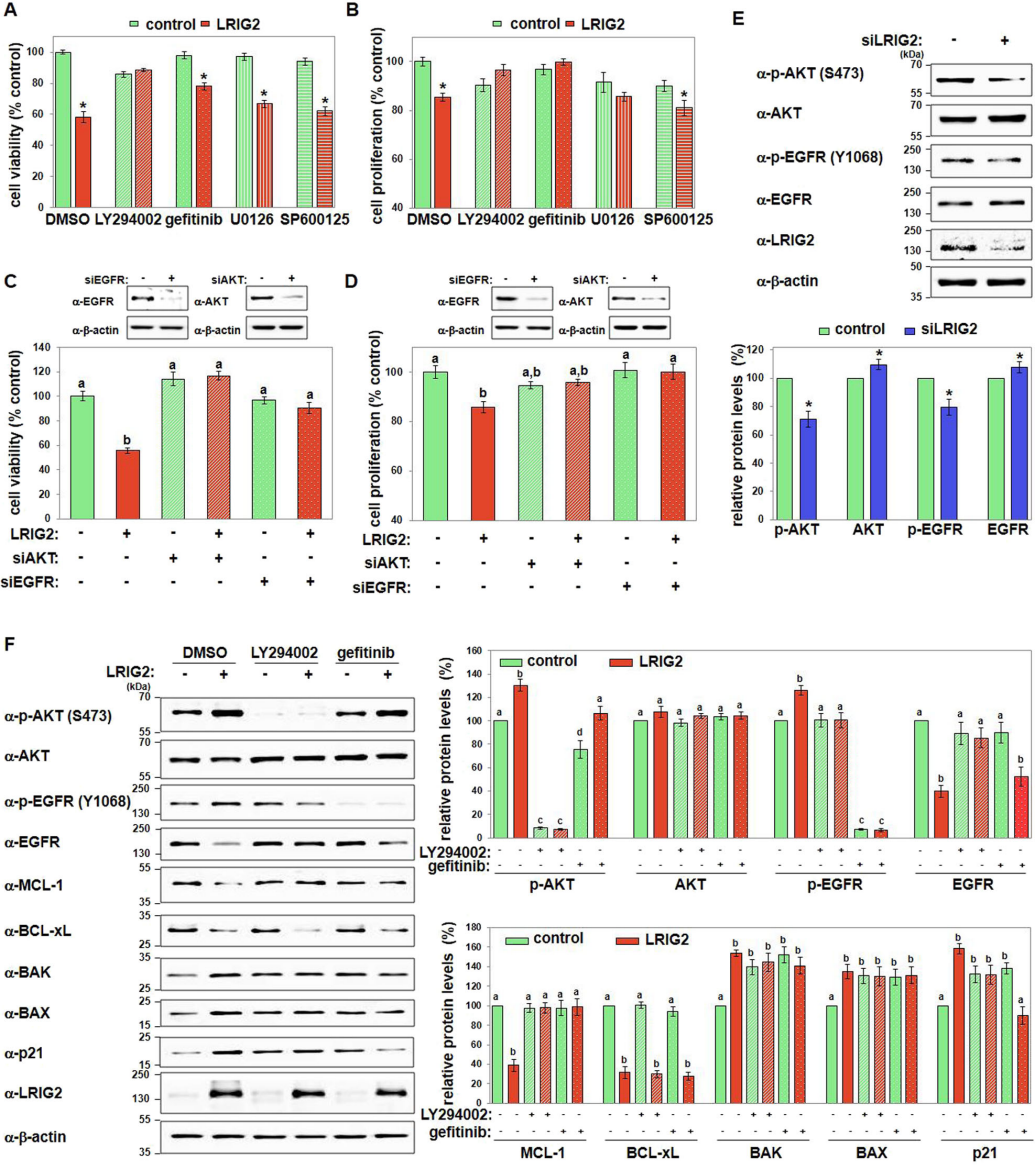
PI3K-mediated phosphorylation of AKT and EGFR by LRIG2 mediates the growth-inhibitory effects of LRIG2. **a**, **b** LRIG2-overexpressing Hec-1A cells were treated with DMSO (0.1%), LY294002 (30 µM), gefitinib (10 µM), U0126 (10 µM), or SP600125 (10 µM) for 24 h, and cell viability **a** and cell proliferation **b** were assayed. Results are mean ± SEM of three independent experiments performed in triplicates. **c**, **d** Hec-1A cells were transfected with control siRNAs, AKT-specific siRNA, or EGFR-specific siRNAs for 24 h, followed by LRIG2 overexpression for additional 24 h. Then, cell viability **c** and cell proliferation **d** were determined. **e** Hec-1A cells were transfected with siRNA #1 for LRIG2. Representative immunoblots and quantified results of three independent experiments for the indicated kinases are shown. **f** Cells presented in **a** were used. Representative immunoblot results and quantified data (mean ± SEM) from three independent experiments are shown. Asterisks and different letters indicate statistically significant values (*p* < 0.05)

To discover the signaling pathway(s) that mediate LRIG2-induced growth inhibition, we used inhibitors of key kinases. We found that the phosphoinositide 3-kinase (PI3K) inhibitor LY294002 or the EGFR inhibitor gefitinib completely or partially prevented LRIG2-induced apoptosis, respectively (Fig. 4a). In contrast, inhibition of extracellular signal-regulated kinase (ERK) or c-Jun N-terminal kinase (JNK) using U0126 or SP600125, respectively, did not efficiently prevent LRIG2-induced cell death (Fig. 4a). Both LY294002 and gefitinib also effectively blocked the LRIG2-induced anti-proliferative activity (Fig. 4b). In addition, the LRIG2-induced inhibitory activities on cell survival and proliferation were prevented by the knockdown of AKT or EGFR (Fig. 4c, d), which was analogous with the observations after treatment of AKT or EGFR inhibitors. Thus, we examined the ability of LRIG2 to modulate AKT and EGFR phosphorylation. LRIG2 knockdown decreased phosphorylation of AKT at serine 473 and EGFR at tyrosine 1068 (Fig. 4e). Conversely, LRIG2 overexpression prominently increased AKT and EGFR phosphorylations at Y1068, Y 1086, and Y 1101 (Fig. 4f; Supplementary Figure S2). In addition, we confirm that endogenously expressed LRIG2 and EGFR proteins are associated in endometrial carcinoma cells (Supplementary Fig. S3a), whereas LRIG2 does not regulate *EGFR* mRNA expression (Supplementary Fig. S3b). LRIG2-induced EGFR phosphorylation was efficiently blocked by inhibition of PI3K/AKT, whereas the EGFR inhibitor could not prevent LRIG2-induced AKT phosphorylation (Fig. 4f), suggesting that PI3K acts as an upstream regulator of EGFR activation in LRIG2-mediated signaling. Moreover, LRIG2 failed to downregulate MCL-1 or upregulate BAK, BAX, and p21 when PI3K or EGFR was inhibited (Fig. 4f). In contrast, LRIG2-induced BCL-xL downregulation was not affected by both inhibitors (Fig. 4f), suggesting the implication of alternative signaling pathways. Thus, these results indicate that the LRIG2-induced anti-cancer activities on endometrial carcinoma involve PI3K/AKT and EGFR activation followed by the regulation of MCL-1, BAK, BAX, and p21 expression.

### LRIG2 suppresses endometrial adenocarcinoma growth

**Fig. 5:**
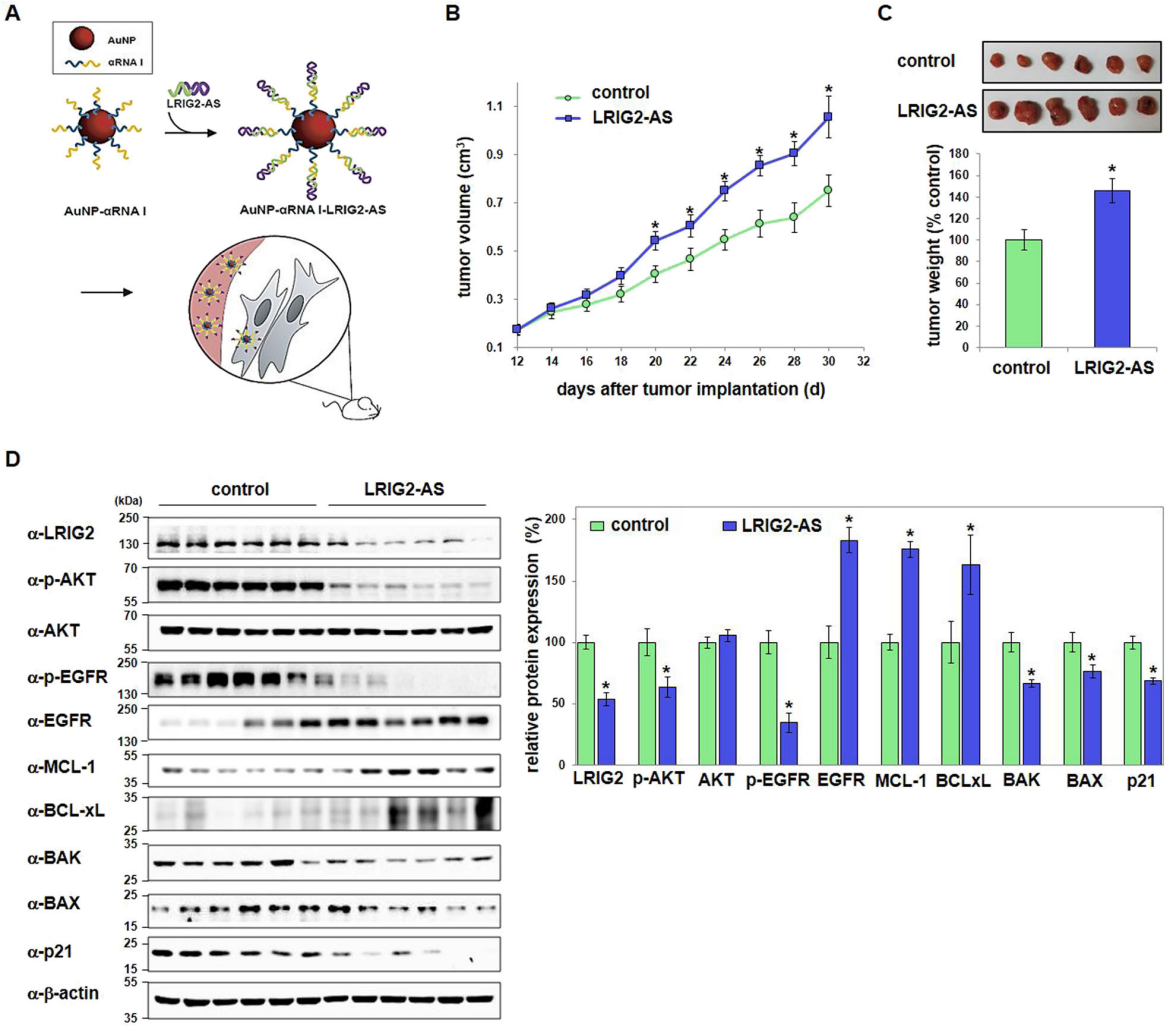
In vivo delivery of LRIG2 antisense DNA stimulates growth of endometrial carcinoma with concomitant regulation of key mediators. **a** A schematic showing generation of functionalized AuNPs conjugated with antisense (AS) DNA specific for LRIG2 or control DNA. **b** Volumes of tumors from mice injected with either the control (*n* = 17) or LRIG2–AS oligonucleotides (*n* = 17) were measured. **c** Pictures of actual tumors and the tumor weights measured 30 d after xenograft implantation are shown. **d** Untreated, control xenografts, or xenografts treated with LRIG2–AS were analyzed and key mediators identified. Representative immunoblots and quantified data for tumors from each group (*n* = 17) are presented. Statistically significant values (mean ± SEM) are labeled with asterisks (*p* < 0.05)

To further confirm the growth-inhibitory functions of LRIG2 in endometrial cancer in vivo, mouse xenotransplantations were performed. A gold nanoparticle (AuNP)-based antisense (AS) oligonucleotide-delivery system we developed previously^16^ was used to deliver LRIG2–AS to silence the endogenous LRIG2 (Fig. 5a). Endometrial adenocarcinomas injected with the AuNP-conjugated LRIG2–AS DNA complex resulted in increased volume and weight of tumors compared with control tumors treated with control DNA (Fig. 5b, c). Efficient knockdown of LRIG2 in xenotransplants was confirmed by immunoblotting (Fig. 5d). In addition, the LRIG2-depleted tumors expressed significantly lower levels of phosphorylated AKT and EGFR accompanied by upregulated EGFR, MCL-1, and BCL-xL, and downregulated BAK, BAX, and p21 (Fig. 5d), altogether akin to in vitro observations (Fig. 4e, f).

### LRIG2 expression is specifically downregulated in endometrial adenocarcinoma patients

**Fig. 6:**
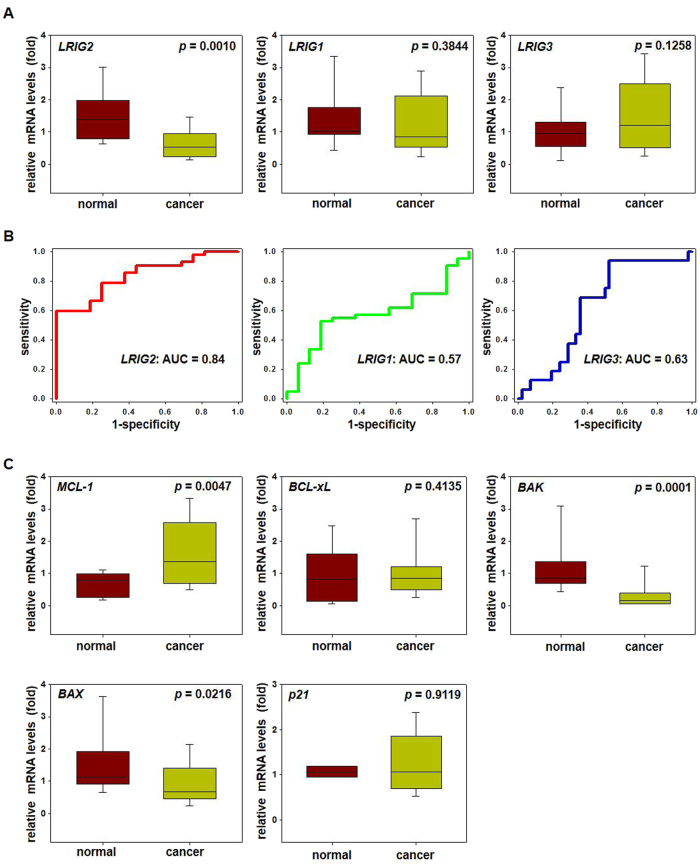
LRIG2 and its downstream mediators are selectively downregulated in endometrial cancer patients. **a** Box-and-whisker plots present relative mRNA expression levels of *LRIG2, LRIG1*, and *LRIG3* in stage 1A endometrial adenocarcinoma tissues of patients (*n* = 42) and control endometrial tissues (*n* = 16). Real-time RT-PCR was performed in triplicates for each specimen, and data were normalized to β-actin. The box plot represents the lower, median, and upper quartiles, and the whiskers represent the 95% confidence interval of the mean. Groups were compared using paired Student’s *t*-tests, and *p* values are presented. **b** ROC curve analyses of *LRIG2*, *LRIG1*, and *LRIG3* mRNA levels are shown with the AUC values. **c** Box-and-whisker plots of key mediators of LRIG2 were prepared and shown as described in **a**

To profile expression of LRIG family of proteins, we examined normal endometrial tissues and stage 1A endometrial adenocarcinoma tissues obtained from patients. Endometrial adenocarcinoma tissues expressed significantly lower levels of *LRIG2* mRNA than controls (Fig. 6a). In contrast, levels of *LRIG2* homologs, *LRIG1* and *LRIG3*, were similar between the two groups (Fig. 6a). Receiver operating characteristic (ROC) curves showed high specificity (0.7381) and sensitivity (0.75) with a cutoff of 0.9088% and an area under the curve (AUC) of 0.84 for *LRIG2* but not for *LRIG1* or *LRIG3* expression (Fig. 6b), suggesting a specific role for LRIG2 in endometrial oncogenesis. In addition, endometrial adenocarcinoma patients expressed significantly higher levels of *MCL-1* and lower levels of *BAK* and *BAX*, whereas *BCL-xL* and *p21* levels did not significantly differ between the two groups (Fig. 6c).

## Discussion

Here, we identified, for the first time, that LRIG2 is downregulated in endometrial adenocarcinoma tissues of patients and functions as a growth suppressor by promoting apoptotic cell death and cell-cycle arrest in Hec-1A and Ishikawa endometrial carcinoma cells. LRIG2 induced mitochondrial apoptotic cell death by regulating the expression of a subset of BCL-2 family of proteins, including MCL-1, BCL-xL, BAK, and BAX both in vivo and in vitro. MCL-1 and BCL-xL are crucial pro-survival proteins required for maintaining mitochondrial integrity in many cell types and preventing the activation of death-effector proteins, BAK and BAX, following heterodimerization with these pro-apoptotic BCL-2 members^17^. Thus, MCL-1 and BCL-xL downregulation concomitant with BAK and BAX upregulation elicited by LRIG2 potently compromised the mitochondrial membrane integrity, resulting in cell death. Especially, inhibition of LRIG2-induced cell death by ectopic expression of MCL-1 or by depletion of either BAK or BAX (Fig. 2d, e) further support our conclusion that LRIG2 induces apoptosis in Hec-1A endometrial carcinoma cells by regulating the delicate equilibrium among the BCL-2 family of proteins.

AKT is an important kinase that governs cellular survival and proliferation^18,19^. Receptor tyrosine kinases, cytokine receptors, G-protein-coupled receptors, and other stimuli activate PI3K, resulting in the production of phosphatidylinositol(3,4,5)-triphosphate that serve as docking sites for proteins such as AKT^20^. Here, we found that PI3K/AKT signaling is a key regulating pathway that mediates the apoptotic and anti-proliferative effects of LRIG2 (Figs. 4 and 5). LRIG2 stimulated PI3K-mediated AKT phosphorylation of serine 473, causing MCL-1 downregulation and upregulation of BAK, BAX, and p21. LRIG2 also activated EGFR by stimulating phosphorylation of its tyrosines 1068, 1086, and 1101, and this response was associated with decreased MCL-1 and increased BAK, BAX, and p21 levels. These results indicate that PI3K/AKT and EGFR pathways are key mediators of the antitumor activities of LRIG2. These observed growth-inhibitory actions of PI3K/AKT and EGFR are surprising, as these pathways are conventionally considered to provide survival-promoting cues for diverse types of cancer cells. In endometrial cancer, the uncontrolled activation of PI3K/AKT is frequently observed, rendering this pathway a promising therapeutic target^2,21,22^. Approximately 30% of endometrioid endometrial cancers harbor mutations or amplification in the catalytic subunit of PI3K, PIK3CA, whereas EGFR overexpression is associated with poor prognosis of endometrial cancer^2,21–23^, and the growth-inhibitory actions of PI3K or EGFR inhibitors on Hec-1A endometrial cancer cells have reported^24–28^. However, our results revealed that phospho-activation of the PI3K and EGFR pathways by LRIG2 contributes to inhibition of endometrial tumor growth, implying a non-canonical role for PI3K/AKT in opposing cell survival and proliferation. These observations suggest that the contribution of PI3K/AKT and EGFR to the pathogenesis and development of endometrial cancer should be assessed with caution and need to be re-evaluated. Furthermore, elucidation of the detailed mechanistic events by which LRIG2 acts as a novel upstream regulator of PI3K/AKT and EGFR in endometrial adenocarcinoma is needed.

LRIG2 is localized on chromosome 1p13 in a region frequently deleted in various types of human cancers^8,29^. The deletion of LRIG2 has been identified in different types of cancer (http://www.cbioportal.org), implying a tumor-suppressive role for LRIG2. Accordingly, our data indicate that LRIG2 acts as a tumor suppressor in endometrial cancer. In glioblastoma, however, LRIG2 reportedly exerts oncogenic effects; LRIG2 knockdown increases apoptosis and decreases proliferation; LRIG2 overexpression stimulates growth of xenotransplanted gliomas; and mice expressing truncated LRIG2 are less prone to develop gliomas induced by platelet-derived growth factor subunit B^30–32^. Thus, LRIG2 functions as either a tumor suppressor or an oncogene likely depending on the cellular context, similar to its well-characterized homolog, LRIG1^5,6^. This study also revealed that measuring the *LRIG2* expression levels can serve as a useful pre-screening molecular biomarker for accurate diagnosis of endometrial adenocarcinoma. Further molecular investigations to decipher the mechanisms underlying LRIG2 downregulation in endometrial adenocarcinoma are needed.

## Material and methods

### Cell culture

Endometrial adenocarcinoma cell lines, Hec-1A (ATCC, Manassas, VA, USA) and Ishikawa (Sigma-Aldrich, St. Louis, MO, USA), were cultured in McCoy’s 5A Modified Medium and Dulbecco’s Modified Eagle’s Medium–Ham’s F12 Medium (DMEM/F12) (Caisson, North Logan, UT, USA), respectively. Wild-type, *bax*^−/−^, *bak*^−/−^, and *bax*^−/−^*bak*^−/−^ mouse embryonic fibroblasts (MEFs) (donated by Dr. C. B. Thompson, University of Pennsylvania, USA) were cultured in DMEM (Caisson), and their authentication was tested by immunoblot analysis. All cell lines were used for experiments within a month of thawing, acquired from the indicted sources between 2005 and 2010, and cell line authentication and routine *Mycoplasma* testing were not performed. Media were supplemented with 10% fetal bovine serum (FBS) and 1% penicillin–streptomycin (Caisson). Cells were grown in 5% CO_2_ at 37 °C.

### Reagents

Anti-LRIG2 (ab121472) was purchased from Abcam (Cambridge, MA, USA). Anti-caspase 3 (9662), anti-caspase 8 (9746), anti-caspase 9 (9502), anti-AKT (9272), anti-pS473-AKT (4058), anti-pY1068-EGFR (3777), and anti-pY1086-EGFR (2220) were purchased from Cell Signaling (Danvers, MA, USA). Antibodies against cytochrome c (sc-7159), EGFR (sc-03), MCL-1(sc-819), BCL-xL (sc-8392), BCL2A1, BCL-2 (sc-65392), BCL-W (sc-6418), BAK (sc-832), BAX (sc-493), BAD (sc-8044), BIM (sc-11425), p21 (sc-397), and β-actin (sc-47778) were purchased from Santa Cruz Biotechnology (Santa Cruz, CA, USA). Anti-pY1101-EGFR (MAB1382) was purchased from Abnova (Taipei, Taiwan). Anti-COX IX (A21347) was purchased from Invitrogen (Carlsbad, CA, USA). LY294002 (L9908) and SP600125 (S5567) were purchased from Sigma. U0126 (662005) was obtained from Calbiochem (San Diego, CA, USA). Gefitinib (G4408) was purchased from LC laboratories (Woburn, MA, USA). Other reagents were purchased from Sigma-Aldrich unless otherwise specified.

### Human subjects and endometrial tissues

Stage 1A endometrial adenocarcinoma tissue samples (*n* = 42) and normal endometrial tissue samples (*n* = 16) from patients were obtained from patients who visited the Seoul Asan Medical Center. Tissue blocks were fixed in formalin and embedded in paraffin (FFPE). FFPE blocks were sectioned (10 μm thick) and each section was assessed by pathologists and analyzed. The study was approved by the Seoul Asan Medical Center Institutional Review Board and performed according to approved guidelines (IRB # 2015-0106).

### Plasmids

The LRIG2– and LRIG2–GFP-encoding plasmids were generous gifts from Dr. Hakan Hedman (Umea University, Sweden)^30^. The human *MCL-1* and *p21* promoters were amplified by polymerase chain reaction (PCR) using the following primers: MCL-1 (F: 5′-ACGACGCGTCCTTGAGGACAGGAGTTG-3′, R: 5′-CTAAAGCTTGGCGAGCAGCTCCTTTAT-3′) and p21 (F: 5′-ACGACGCGTATTGAG AAGCAAAATTGTACT-3′, R: 5′-CTACTCGAGGGGACATGTTCCTGACGCCA-3′). PCR products were digested using *Mlu*Ι (1071A), *Hind*ΙΙΙ (1060A), or *Xho*I (1094A) (Takara Bio, Shiga, Japan) and ligated into pGL3 (Promega, Madison, WI, USA). Cloning of the plasmids encoding the BCL-2 family members was described elsewhere^33,34^.

### RNA interference

To silence LRIG2, two independent small interfering RNAs (siRNAs) targeting different sequences of LRIG2 were used: LRIG2#1 (5′-CUGAUACCGUCAGCCAACA-3′ and 5′-UGUUGGCUGACGGUAUCAG-3′) and LRIG2#2 (5′-CAUCAGCUUGGAAUCACAAAC AUUA-3′ and 5′-UAAUGUUUGUGAUUCCAAGCUGAUG-3′). siRNAs for p21 were 5′-CUUCGACUUUGUCACCGAGUU-3′ and 5′-AACUCGGUGACAAAGUCGAAG-3′. The sequence for the control siRNA was 5′-CCUACGCCACCAAUUUCGU-3′. siRNAs for AKT were 5′-GAAGGAAGUCAUCGUGCCCAA-3′ and 5′-UUGGGCACGAUGACUUCCUUC-3′. siRNAs for EGFR were 5′-GAGGAAAUAUGUACUACGA-3′ and 5′-UCGUAGUACAUAUUUCCUC-3′. All siRNAs were purchased from Bioneer (Daejeon, Korea). Sense and antisense oligonucleotides were annealed in the annealing buffer (Bioneer).

### Transfection

Cells were transfected using a MicroPorator MP-100 (Invitrogen) as described previously^35^.

### RNA extraction and real-time RT-PCR

Isolation of total RNAs and real-time RT-PCR were performed as described^36^. Primer sequences used are detailed in Supplementary Table S1.

### Cell-viability assays

Cell viability was assayed as previously described^36^.

### Apoptosis analysis

Apoptotic cells positive for annexin-V were detected as previously reported^35^.

### Western blotting

Western blotting was performed as previously described^36^.

### Cytochrome *c* release

Release of cytochrome *c* into the cytoplasm was detected using a digitonin-based method as described previously^35^.

### Luciferase reporter assay

Luciferase assays were performed as described previously^35^.

### Cell-cycle analysis

Cell-cycle was analyzed as described previously^37^.

### Cell-proliferation assay

Cell proliferation was assayed using the 5′-bromo-2′-deoxyuridine labeling and detection kit ΙΙΙ (11 444 611 001) (Roche, Mannheim, Germany)^38^.

### Immunoprecipitation analysis

Immunoprecipitation analysis was performed as described previously^38^.

### Preparation of functionalized gold nanoparticle (AuNP)-conjugated with antisense DNAs

Functionalized gold nanoparticles (AuNP–αRNA I) were prepared using citrate-stabilized gold nanoparticles (15 nm) (EM.GC 15) (BBI Life Science, Cardiff, UK) conjugated with RNA I oligonucleotide (αRNA I) according to previously described procedures^16,39^. The antisense (AS) oligonucleotide for LRIG2 was 5′-CGCTAGCAGAGCCGAGATTGTTGGCTGACGGTATCAG-3′ and the control oligonucleotide was 5′-CGCTAGCAGAGCCGAGATACGAAATTGGTGGCGTAGG-3′. Common αRNA I sequences are underlined.

### Mouse xenotransplantation

Hec-1A cells (~2 × 10^6^) were subcutaneously injected into 6-week-old BALB/c nu/nu immunodeficient mice (ORIENT BIO, Seongnam, Korea), whose weights ranged between 18 and 20 g. We randomly allocated mice to two groups. 12 days after xenotransplantation, AuNP–αRNA I control or AuNP–αRNA I–AS LRIG2 composites, suspended in PBS, were injected into the tumor sites every 2 days. Mice were weighed and sizes of the tumors were measured every other day. The volume (mm^3^) of each tumor ((length × width^2^ × *π*)/6) was determined over a 4-week period after xenotransplantation. Tumor-bearing mice were killed 18 days after the first injection of the functionalized AuNP composites, and tumors were excised and weighed. Animal guidelines were approved by the Chung-Ang University Institutional Animal Care and Use Committee (IRB# CAU2012-0044), and the animals were treated as described in the protocol.

### Statistical analyses

Multiple-comparison analyses of values were performed using the Student–Newman–Keuls test, and Student’s *t*-test was used for comparisons with controls using SAS version 9.2 (SAS Institute, Cary, NC, USA) and SigmaPlot (Systat Software, San Jose, CA, USA). The data are presented as mean ± SEM. *p* < 0.05 was considered statistically significant.

## Electronic supplementary material

Supplementary data
