# Supplementary material for: LRIG2 is a growth suppressor of Hec-1A and Ishikawa endometrial adenocarcinoma cells by regulating PI3K/AKT- and EGFR-mediated apoptosis and cell-cycle
Source: Oncogenesis. 2018 Jan 23;7(1):3. doi: 10.1038/s41389-017-0019-1 (PMC5833696; doi:10.1038/s41389-017-0019-1)
Supplement: Supplementary file 1 — Supplementary data [file 41389_2017_19_MOESM1_ESM.docx]

**Supplementary Table S1. Oligonucleotide sequences of primers**

| Gene | Sequence |
| --- | --- |
| *MCL-1* | F : 5′-TGCTTCGGAAACTGGACATCA-3′ |
|  | R : 5′-TAGCCACAAAGGCACCAAAAG-3′ |
| *BCL-xL* | F : 5′-AGGGACTGAATCGGAGATGG-3′ |
|  | R : 5′-CCTCAGCGCTTGCTTTACTG-3′ |
| *BCL2A1* | F : 5′-CTATCTGCAGTGCGTCCTAC-3′ |
|  | R : 5′-TTCAAATGCAAATATGGTTA-3′ |
| *BCL-2* | F : 5′-GTGGAGGAGCTCTTCAGGGA-3′ |
|  | R : 5′-AGGTGCCGGTTCAGGTACTC-3′ |
| *BCL-w* | F : 5′-AGATGAGTTCGAGACCCGCT-3′ |
|  | R : 5′-CCAGTGGTTCCATCTCCTTGTTG-3′ |
| *BAK* | F : 5′-CTGCAACCTAGCAGCACCAT-3′ |
|  | R : 5′-TGCTGGTAGACGTGTAGGGC-3′ |
| *BAX* | F : 5′-CCCTTTTGCTTCAGGGTTTC-3′ |
|  | R : 5′-GCCACTCGGAAAAAGACCTC-3′ |
| *BAD* | F : 5′-CCAGAGTTTGAGCCGAGTGA-3′ |
|  | R : 5′-GTAGGAGCTGTGGCGACTCC-3′ |
| *BIM* | F : 5′-TAAGTTCTGAGTGTGACCGAGA-3′ |
|  | R : 5′-GCTCTGTCTGTAGGGAGGTAGG-3′ |
| *p21* | F : 5′-TGTCCGTCAGAACCCATGC-3′ |
|  | R : 5′-AAAGTCGAAGTTCCATCGCTC-3′ |
| *LRIG2* | F : 5′-AAATGCAGCGGAATGGAATTAGC-3′ |
|  | R : 5′-CCCCTTGTTTACTCGTGTAAGGT-3′ |
| *LRIG1* | F : 5′- GGACTTGCCGAACCTACAGG-3′ |
|  | R : 5′- GCTGCGAATCTTGTTGTGCTG-3′ |
| *LRIG3* | F : 5′- ACCATCCGAGCTGGGGCCAT-3′ |
|  | R : 5′- ACCATCCGAGCTGGGGCCAT-3′ |
| *EGFR* | F : 5′- TTGCCGCAAAGTGTGTAACG-3′ |
|  | R : 5′- GTCACCCCTAAATGCCACCG-3′ |
| *β-actin* | F : 5′-GCCCTGGCACCCAGCACAAT-3′ |
|  | R : 5′- ATGCGTCTCTCCCGTGCAGC-3′ |

**Supplementary Figure S1**

**
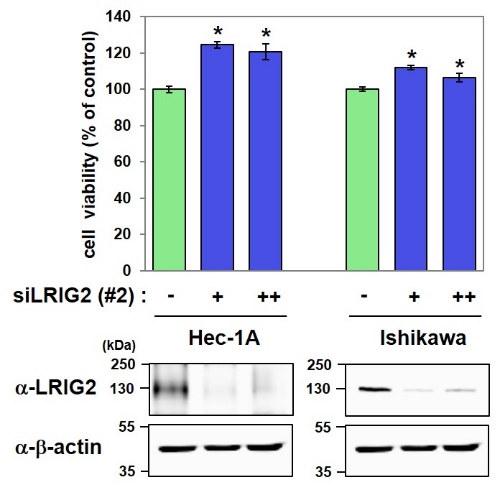
a b**

**
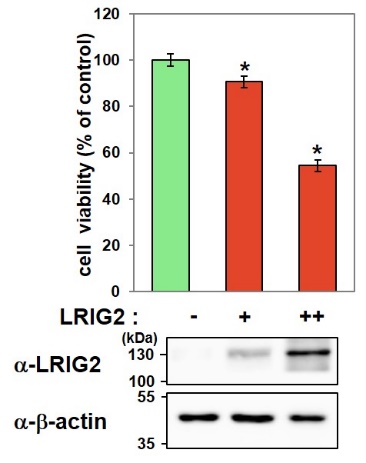
**

**Supplementary Figure S1. LRIG2 is a cell death protein in endometrial carcinoma cells**. (**a**) Hec-1A cells were transfected with 100 ng of an empty vector or non-tagged LRIG2-expressing plasmid. Cell viability was analyzed 24 h after transfection. (**b**) Hec-1A and Ishikawa cells were transfected with siRNA #2 (100 or 200 nM) targeting LRIG2. As a control, scrambled siRNAs (200 nM) were transfected. Cell viability was analyzed 24 h after transfection. Efficient knock down of LRIG2 was demonstrated by immunoblotting. All quantified results are mean ± SEM of three independent experiments performed in triplicates. Asterisks indicate statistically significant values compared to the control (*p* < 0.05).

**Supplementary Figure S2**


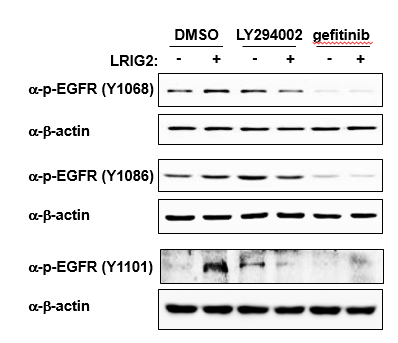


**Supplementary Figure S2. LRIG2 stimulates phosphorylations of EGFR at Y1068, Y1086, and Y1101 residues.** LRIG2-overexpressing Hec-1A cells were treated with DMSO (0.1%), LY294002 (30 µM), or gefitinib (10 µM) for 24 h. The cell lysates were analyzed by immunobloting using specific antibodies that recognize respective phosphorylated residues.

**Supplementary Figure S3**


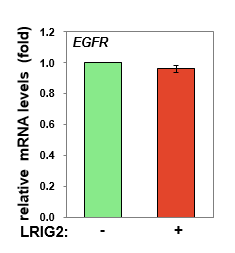
**a b**


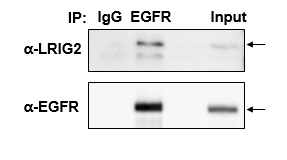


**Supplementary Figure S3. LRIG2 interacts with EGFR in endometrial carcinoma cells**. (**a**) The interaction between endogenous LRIG2 and EGFR proteins was determined in Hec-1A cells after immunoprecipitation with control IgG or an anti-EGFR antibody. (**b**) Hec-1A cells were transfected with 3 μg of an empty vector or LRIG2-expressing plasmid. Change in the mRNA level of the *EGFR* in Hec-1A cells were quantified by real-time PCR.
